# Spatiotemporal analysis of genetic perturbations reveals a genetic cascade driving *Tribolium* gap gene initialization

**DOI:** 10.1242/bio.062391

**Published:** 2026-01-19

**Authors:** Mahla Ahmadi, Heike Rudolf, Christine Mau, Jimena Garcia-Guillen, Ezzat El-Sherif

**Affiliations:** ^1^ School of Integrative Biological and Chemical Sciences (SIBCS), University of Texas Rio Grande Valley (UTRGV), Edinburg, TX 78539, USA; ^2^ Division of Developmental Biology, Department of Biology, Friedrich-Alexander-Universität Erlangen-Nürnberg, 91054 Erlangen, Germany; ^3^ Departmen of Evolutionary Developmental Genetics, Georg-August-University Göttingen, 37077 Göttingen, Germany

**Keywords:** Temporal patterning, *Tribolium*, Segmentation, Gene expression waves, Gap genes, Gene regulatory network

## Abstract

The ‘French flag’ model has long served as the prevailing framework for explaining how morphogen gradients generate spatial domains during embryonic development. However, recent evidence indicates that many tissues establish patterns by translating the sequential activation of genes into spatial domains. While the sequential nature of this process is becoming clear, the mechanisms that mediate these temporal dynamics and translate them into stable spatial boundaries remain debated. Using the gap gene network in the flour beetle *Tribolium castaneum* [which mediates the regionalization of the anterior-posterior (AP) axis into different axial fates through the regulation of downstream Hox genes] as a model, we combined hybridization chain reaction *in situ* hybridization, parental RNA interference (RNAi), and computational modeling to dissect these mechanisms. Our high-resolution spatiotemporal analysis indicates that gap genes initially function as a genetic cascade in the posterior growth zone. Specifically, RNAi perturbations reveal that the disruption of upstream genes prevents the initiation of downstream targets in the posterior rather than merely affecting their anterior maintenance. Conversely, the knockdown of downstream repressors leads to the posterior persistence of upstream genes. Furthermore, we investigated the relationship between this dynamic initiation phase and anterior maintenance. We observe that in *milles-pattes* (*mlpt*) RNAi embryos, the gap gene *shavenbaby* (*svb*) fails to propagate anteriorly out of the growth zone, indicating that the anterior maintenance of *svb* is actively mediated by other genes in the network. Computational simulations demonstrate that a gene network switching framework, where regulatory interactions reconfigure across the AP axis, successfully reproduces these complex phenotypes. These findings provide definitive spatiotemporal evidence that *Tribolium* gap gene initialization is driven by a genetic cascade, and support a model in which dynamic network rewiring converts this cascade into stable spatial patterns more anteriorly.

## Teaser

**Summary:** Embryonic patterning can arise from temporal gene activation translated into space; this study shows that *Tribolium* anterior-posterior patterning follows this logic at the genetic level.

## INTRODUCTION

Embryogenesis is an intricate process wherein a single fertilized egg undergoes extensive cell divisions and differentiations to form a complete organism. A pivotal step in this process is the establishment of cell identities, where individual cells adopt specific functions and occupy a precise location within the embryo. Understanding gene regulatory networks (GRNs) that regulate these cell identities is vital for comprehending how organisms develop their body plans and structures (Levine and Davidson, 2005; Davidson, 2010). These networks involve the activation and interaction of specific genes that define cell fate in response to positional information, ensuring proper tissue and organ formation during development.

One widely used model to explain body patterning during embryogenesis is the French flag model (Wolpert, 1969). According to this model, cells read the concentration of morphogens, and, depending on whether the concentration crosses certain thresholds, they activate different genes. This creates sharp boundaries between gene expression domains, helping cells know their position and fate within the developing embryo. While this model has been foundational, it assumes that morphogen gradients are stable and produce precise thresholds. Recent studies, however, show that these gradients are often noisy and dynamic (Irizarry and Stathopoulos, 2021; Sandler and Stathopoulos, 2016), challenging the idea that fixed concentration thresholds alone can reliably direct consistent gene expression during development.

**Fig. 1.**
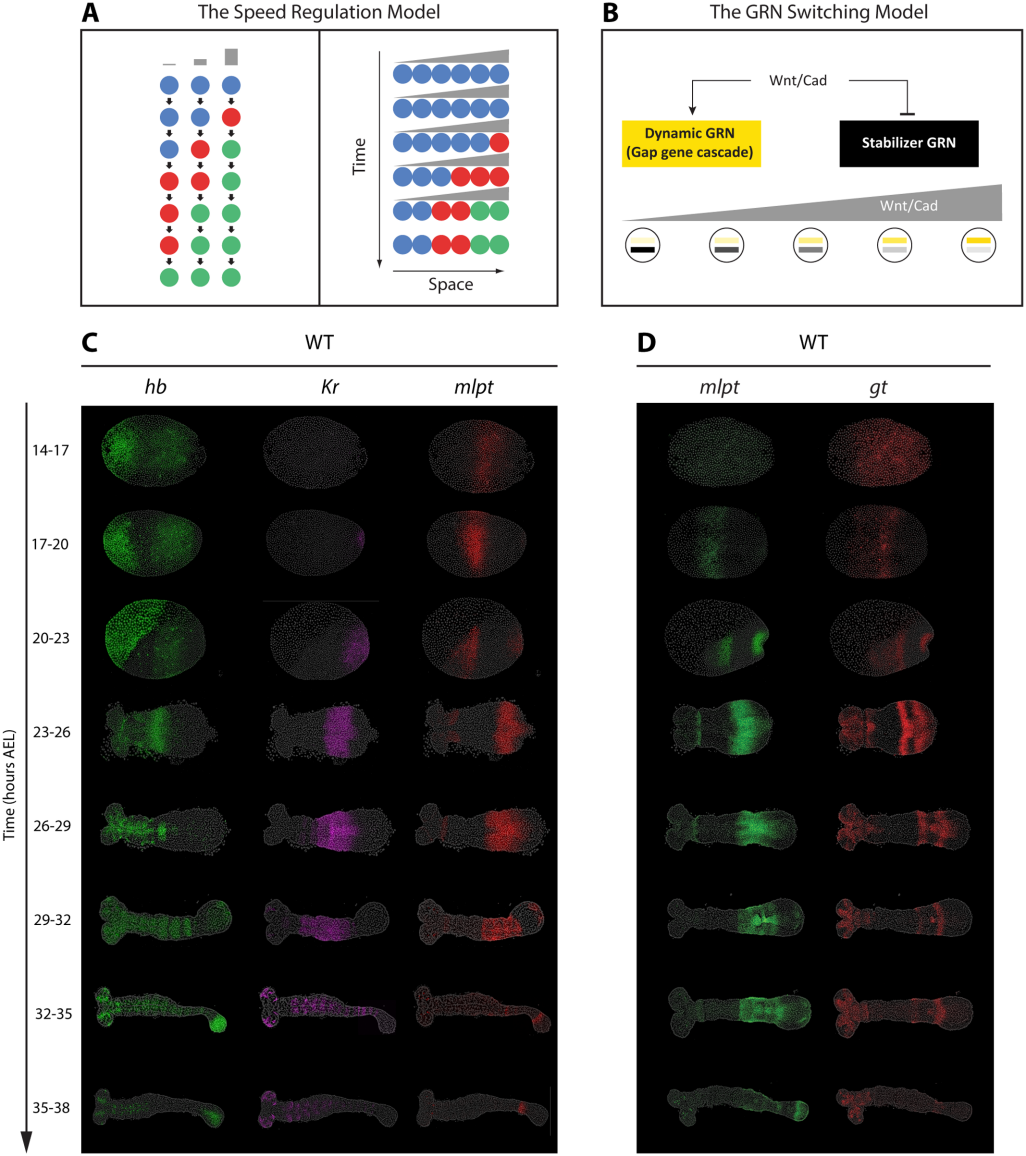
**Models of gap gene regulation and spatiotemporal dynamics of gap gene expression in WT *Tribolium* embryos.** (A) The speed regulation model. In this framework, a morphogen gradient modulates the speed of a temporal sequence of gene activations, such that sequential waves of expression are progressively frozen into spatial domains. (B) The GRN switching model. Here, high morphogen gradient (e.g. Wnt/Cad in the case of gap gene regulation in *Tribolium*) levels activate a dynamic GRN in the posterior, organized as a genetic cascade, while lower morphogen levels favor a static stabilizer GRN that locks gene expression into stable domains. (C,D) HCR FISH detecting subsets of the four core gap genes (*hb*, *Kr*, *mlpt*, *gt*) in staged WT embryos (14-38 h AEL). Panel C shows expression of *hb*, *Kr*, and *mlpt* concurrently in the same embryos, while panel D shows *mlpt* and *gt* concurrently in the same embryos. All embryos are oriented with anterior to the left and posterior to the right.

The speed regulation model (Fig. 1A) (Zhu et al., 2017; Diaz-Cuadros et al., 2021) offers an alternative to the French flag model by focusing on the timing of gene activation rather than fixed morphogen concentration thresholds. In this model, each cell undergoes successive activations of genes, with a morphogen (referred to as the ‘speed regulator’) controlling the rate at which these activations occur (Fig. 1A, left). Specifically, the model uses a morphogen gradient to modulate the speed of gene activation across cells: high morphogen concentrations trigger rapid gene activation, while lower concentrations slow it down. This creates (the appearance of) waves of gene expression that propagate from regions of high morphogen concentration to lower ones, eventually stabilizing spatial expression domains as the gradient declines (Fig. 1A, right).

This type of temporal modulation has been implicated in patterning various embryonic tissues, like the segmentation of anterior-posterior (AP) axis in arthropods and vertebrates (Diaz-Cuadros et al., 2021; Palmeirim et al., 1997; Dale and Pourquié, 2000; El-Sherif et al., 2012, 2014; Sarrazin et al., 2012; Stollewerk et al., 2003; Kanayama et al., 2011; Chipman and Akam, 2008; Peel et al., 2005), Hox gene regulation in vertebrates (Diaz-Cuadros et al., 2021; Deschamps and Duboule, 2017; Gaunt, 2001) and gap gene regulation in insects (Zhu et al., 2017; Boos et al., 2018; Rudolf et al., 2020), and the patterning of ventral neural tube (Balaskas et al., 2012; Dessaud et al., 2007) and limb bud (Harfe et al., 2004; Towers and Tickle, 2009) in vertebrates. However, while the speed regulation model explains the general mechanism of body patterning, it does not provide detailed molecular mechanisms of as how the timing of a GRN is modulated by a morphogen gradient. Understanding of this conversion from temporal to spatial expression is essential for understanding the gene regulatory mechanisms driving patterning during development.

To answer this question, we focus in this study on the gap gene network in the flour beetle *Tribolium castaneum*, which offers a valuable system for understanding GRN dynamics during embryonic patterning. Gap genes were specifically selected because previous studies suggest their expression patterns closely follow the principles proposed by the speed regulation model (Zhu et al., 2017; Diaz-Cuadros et al., 2021; Boos et al., 2018; Rudolf et al., 2020; Verd et al., 2018, 2017; Clark et al., 2019; Clark and Peel, 2018). Specifically, gap genes *hunchback* (*hb*) (Wolff et al., 1995; Marques-Souza et al., 2008), *Krüppel* (*Kr*) (Cerny et al., 2005), *milles-pattes* (*mlpt*) (Savard et al., 2006), and *giant* (*gt*) (Bucher and Klingler, 2004) exhibit sequential waves of expression propagating from posterior to anterior regions, mirroring the temporal progression predicted by speed regulation (Zhu et al., 2017). According to this framework, a posterior-to-anterior gradient, namely Caudal (Cad) and/or Wnt signaling, controls the timing of gap gene activation, converting a temporal sequence into a spatial pattern (Zhu et al., 2017). Supporting this, RNA interference (RNAi) experiments targeting the Wnt pathway cause specific shifts in both the timing and positioning of gap gene expression that align with speed regulation predictions (Zhu et al., 2017). However, the specific mechanism by which the Wnt/Cad gradient modulates gap gene dynamics remains an open question.

Two hypotheses have been proposed (Zhu et al., 2017; Boos et al., 2018; Garcia-Guillen and El-Sherif, 2025): the ‘general kinetic modulation’ model and the ‘GRN switching’ model. Crucially, both models rely on the premise that gap genes are wired into a genetic cascade that drives their sequential activation. The ‘general kinetic modulation’ model posits that the morphogen gradient modulates global kinetic factors (e.g. transcription and decay rates) to control the speed of the genetic cascade without altering its wiring (Garcia-Guillen and El-Sherif, 2025; Manser and Perez-Carrasco, 2024). The ‘GRN switching’ model suggests that the morphogen modulates the network topology itself (Fig. 1B). In this scenario, high morphogen levels in the posterior activate a dynamic GRN (the genetic cascade driving sequential activation), while lower levels in the anterior trigger a switch to a static GRN (a stabilizing network that locks expression into domains) (Fig. 1B) (Zhu et al., 2017; Garcia-Guillen and El-Sherif, 2025). In this framework, the morphogen gradient regulates the balance between dynamic and static GRN architectures, thereby determining the tempo of the system. At high morphogen levels, the dynamic GRN (which manifests as a genetic cascade in *Tribolium*) operates at a maximal rate. Conversely, at lower concentrations, the static GRN exerts an antagonistic influence, effectively slowing the timing of the dynamic cascade.

We note here that verifying the existence of a genetic cascade during the initialization phase is a prerequisite for validating either model. To date, the genetic cascade hypothesis has rested largely on two lines of evidence: the sequential appearance of gap genes in wild-type (WT) embryos (Zhu et al., 2017; Boos et al., 2018), and knockdown phenotypes that appear consistent with cascade-like regulation (Wolff et al., 1995; Marques-Souza et al., 2008; Cerny et al., 2005; Savard et al., 2006; Bucher and Klingler, 2004; Marques-Souza, 2007). Notably, these knockdown phenotypes were not originally interpreted as evidence of a genetic cascade; this hypothesis was only proposed after documenting that gap genes are activated sequentially in the posterior growth zone (Zhu et al., 2017). However, because these previous studies did not investigate the process in the temporal dimension, relying instead on static, late-stage expression patterns after domain stabilization, it remains unclear whether the reported phenotypes merely resemble the expected outcomes of a genetic cascade or are, in fact, driven by one.

To resolve this, we employed hybridization chain reaction (HCR) *in situ* hybridization (Choi et al., 2018), parental RNAi, and computational modeling to rigorously test the genetic cascade hypothesis. By tracking gap gene expression in space and time across WT and perturbed embryos, we provide strong evidence that gap genes are indeed wired into a genetic cascade during the initialization phase in the posterior. Specifically, we show that the disruption of a gap gene prevents the initiation of subsequent genes in the sequence while causing the overexpression of the preceding gene, confirming the wiring logic shared by both theoretical models.

Furthermore, our data provide insights that help distinguish between the two proposed mechanisms of stabilization. We examined the dynamics of *svb*, which functions together with *mlpt* as a gap gene in *Tribolium* (Ray et al., 2019). We find that *svb* is initiated in the posterior as part of the dynamic cascade but requires specific genetic inputs to stabilize anteriorly. In *mlpt* RNAi embryos, *svb* fails to propagate anteriorly and remains restricted to the posterior growth zone. This phenotype is difficult to explain via simple kinetic modulation but is fully consistent with the GRN switching model, where *mlpt* is required to transition *svb* from the dynamic posterior cascade to a static anterior maintenance mode. Together, our findings confirm the cascade-driven initialization of the AP axis and support a model where network switching mediates the transition from temporal dynamics to stable spatial patterns.

## RESULTS

### Spatiotemporal dynamics of gap gene expression in WT *Tribolium* embryos

The spatiotemporal dynamics of gap gene expression in *Tribolium* embryos have previously been described (Zhu et al., 2017; Marques-Souza et al., 2008; Cerny et al., 2005; Savard et al., 2006; Bucher and Klingler, 2004); however, we document them here again using HCR *in situ* hybridization to directly compare WT expression patterns with those observed following RNAi knockdown of specific gap genes (Fig. 1). In general, gap genes in *Tribolium* are expressed in a sequential manner, forming dynamic waves that originate in the posterior and propagate toward the anterior during development. To capture these dynamics, we analyzed gene expression at 3 h intervals from 14-17 h after egg lay (AEL) through 35-38 h AEL (Fig. 1C,D).

During the blastoderm stages (14-17, 17-20, 20-23 h AEL), zygotic *hb* initiates in the serosa and posterior, then clears from the posterior to leave a stable anterior domain (Fig. 1C, green). During the germband phase (23-26 to 32-35 h AEL), the posterior remains devoid of *hb* until 32-35 h, when posterior expression reappears and persists through AP patterning (Fig. 1C, green). *Kr* emerges at 17-20 h AEL at the posterior of the blastoderm, clears from posterior by 23-26 h AEL leaving an expression domain more anteriorly, and remains absent from the posterior thereafter (Fig. 1C, purple). *mlpt* expression begins at 20-23 h AEL in the posterior, clears between 23-26 and 26-29 h leaving an anterior domain, and at 29-32 h is re-expressed posteriorly but fades rapidly to a narrow posterior stripe (Fig. 1C, red; Fig. 1D, green). Trunk expression if *gt* is first detectable in the posterior at 20-23 h AEL undergoes rapid clearing and reappearing, resulting in two distinct stripes by 26-29 h AEL (Fig. 1D, red). All resolved gap gene expression patterns at the anterior persist for a while before eventually fading, except for the most posterior late expression of *hb*, which stays restricted to the growth zone. Some genes are also activated in additional domains that are not considered in this study. For example, *hb* and *Kr* are expressed in the central nervous system (CNS) of anterior germbands (*hb* from 29 to 32 h AEL onward, and *Kr* from 32 to 35 h AEL onward). In addition, *hb* is expressed in the serosa, while *mlpt* and *gt* are activated in the head primordia (Fig. 1C,D).

Taken together, these gene expression patterns illustrate a general principle of *Tribolium* gap gene regulation: gap genes are activated sequentially in dynamic waves that initiate in the posterior and move anteriorly. This posterior-to-anterior propagation underlies the temporal unfolding of the body plan during early embryogenesis.

### Knockdown phenotypes of gap genes suggest that they are wired into a genetic cascade

The sequential activation of gap genes raises the possibility that these genes may be organized into a genetic cascade. Previous studies describing various *Tribolium* gap gene knockdown phenotypes (Cerny et al., 2005; Savard et al., 2006; Bucher and Klingler, 2004; Marques-Souza, 2007) have previously led us to the conclusion that gap genes might indeed function as a genetic cascade (Zhu et al., 2017). However, those previous observations provided only static snapshots at specific developmental stages, lacking detailed temporal resolution. Without dynamic information, phenotypes observed in knockdown embryos might only superficially resemble those expected from a genetic cascade, rather than truly reflecting sequential genetic interactions. Because a genetic cascade inherently unfolds over time, temporal information is essential to rigorously test the genetic cascade hypothesis.

Therefore, we systematically tracked the spatiotemporal dynamics of gap gene expression following individual gap gene knockdowns, using carefully staged egg collections. Below, we describe how the sequential activation of gap genes and the associated propagating waves are affected by specific gap gene disruptions.

In a genetic cascade, each gene's expression is typically expected to mediate activation of the subsequent gene, while simultaneously facilitating the repression of preceding genes. Notably, this cascade arrangement does not necessarily imply direct positive activations. A cascade can also be composed entirely of inhibitory interactions, with sequential activation arising indirectly through de-repression (i.e. repression of repressors). Regardless of the exact mechanism, both genetic cascade configurations predict that knocking down one gene should disrupt the expression of genes that are normally activated later in the cascade, while causing persistent or expanded expression of genes that act earlier in the sequence. Here, we directly test these predictions by examining gap gene expression dynamics across both space and time following targeted RNAi knockdowns.

#### *hb* RNAi disrupts downstream gap gene expression

**Fig. 2.**
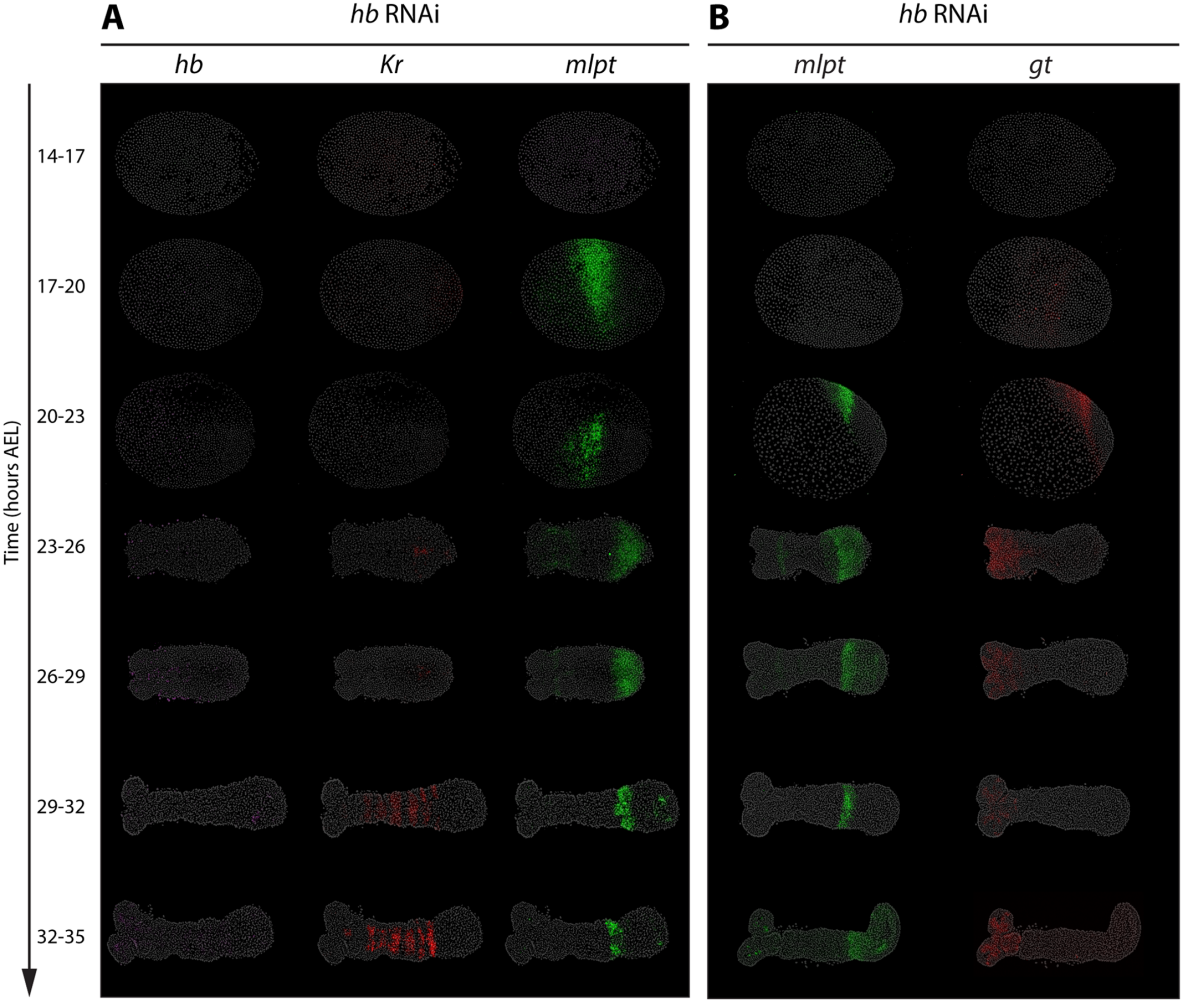
***hb* RNAi disrupts downstream gap gene expression.** Panels A and B show HCR FISH detecting subsets of the core gap genes (*hb*, *Kr*, *mlpt*, *gt*) in staged in *hb* RNAi embryos (14-35 h AEL). Panel A displays the expression patterns of *hb*, *Kr*, and *mlpt* in the same embryos, while Panel B shows the expression of *mlpt* and *gt* in the same embryos*.* All embryos are oriented with anterior to the left and posterior to the right.

To examine the role of *hb* within the gap GRN, we performed parental RNAi targeting *hb* and analyzed embryos from 14-17 to 32-35 h AEL (Fig. 2). In *hb* RNAi embryos, *hb* expression was absent or strongly reduced at all stages examined, confirming efficient knockdown (Fig. 2A).

Consistent with *hb* acting at the top of the gap cascade, the trunk expression of subsequent gap genes in the cascade were mostly diminished: *Kr* (Fig. 2A, red; except for the late striped *Kr* expression in the CNS), most of *mlpt* expression (Fig. 2A,B, green), and *gt* (Fig. 2B, red). The notable exception is the expression of *mlpt*. A small anterior subdomain of the early posterior *mlpt* domain persisted in *hb* RNAi embryos and thus appears to activate independently of other gap inputs (Fig. 2A,B, green).

Notably, Kr, (most of) *mlpt*, and *gt* expression in *hb* knockdowns fail to initiate in the posterior, rather than appearing and later being lost after anterior stabilization. This supports the view that gap genes are wired into a genetic cascade in the posterior, where high Cad levels drive their sequential activation, and highlights *hb* as the initiating gene required to trigger this cascade during early embryonic patterning.

#### RNAi knockdown of *Kr* results in the expansion of *hb* expression domain and reduction of *mlpt* and *gt* expression

**Fig. 3.**
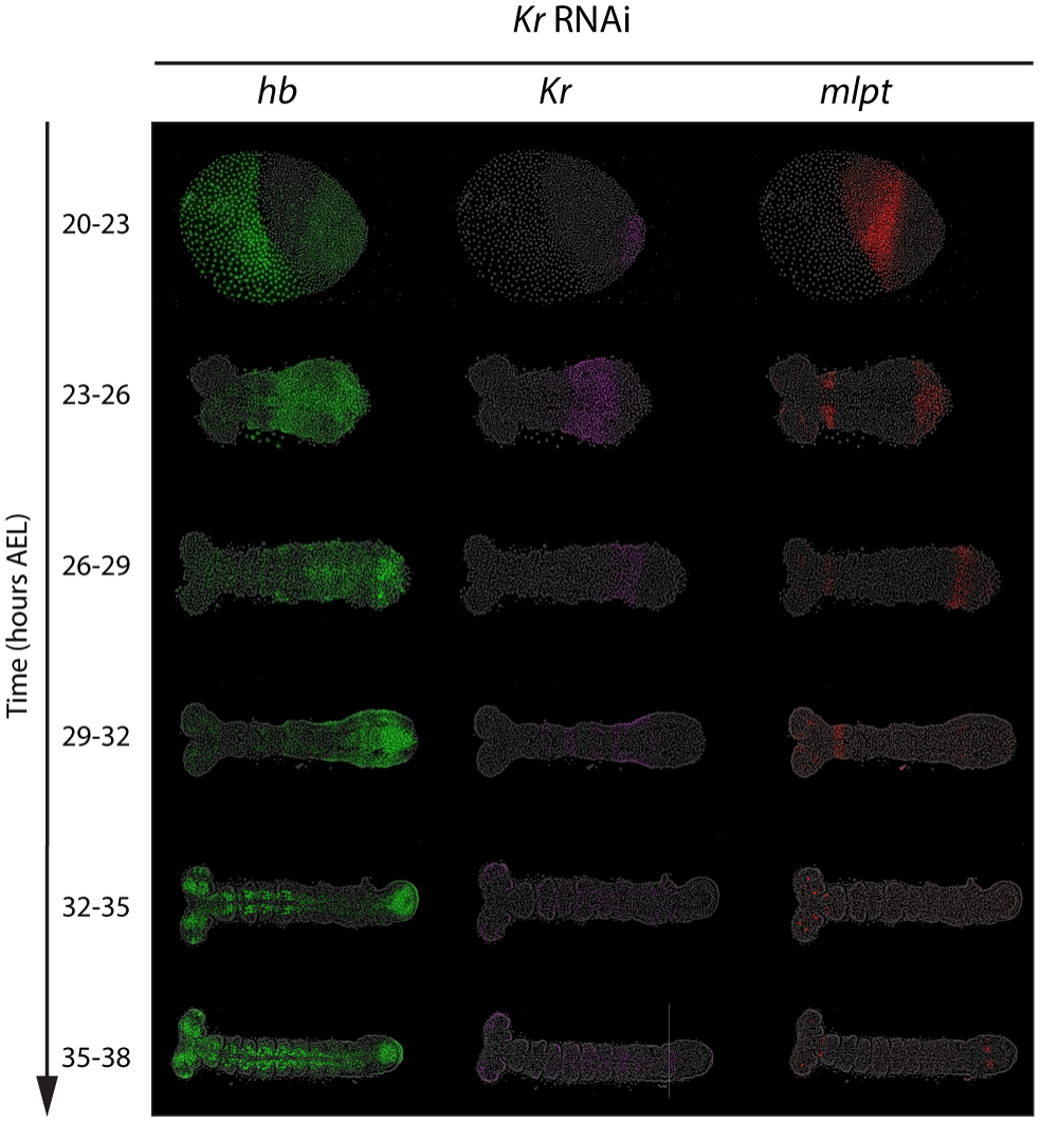
***Kr* RNAi expands *hb* expression domain and reduces *mlpt* expression.** HCR FISH concurrently detecting subset of the core gap genes (*hb*, *Kr*, *mlpt*) in staged embryos (20-38 h AEL) following parental *Kr* RNAi. All embryos are oriented with anterior to the left and posterior to the right.

In embryos subjected to *Kr* RNAi, *Kr* expression was indistinguishable from background levels in most embryos, confirming effective knockdown (Fig. 3). Under these conditions, *hb* expression persisted and failed to clear from the posterior, consistent with *Kr* normally repressing its upstream gene *hb* (Fig. 3, green). Conversely, genes that normally act later in the cascade showed severely reduced expression. The *mlpt* domain was either strongly diminished or completely absent, except for a small anterior region of expression, which again appears to be activated independently of earlier genes in the cascade (Fig. 3, red), while *gt* expression is greatly reduced or absent (Fig. S1, red).

Notably, genes that act later in the cascade than *Kr* failed to initiate altogether, rather than first appearing posteriorly and then disappearing after their stabilization in anterior domains. At the same time, the earlier gene, *hb*, remained persistently expressed in the posterior upon *Kr* knockdown, instead of being cleared from the posterior initially and then expanding toward the posterior after stabilization. These results confirm the regulatory role of *Kr* within the gap GRN and align with a gap genetic cascade model active in the posterior, where each gene both promotes the activation of the next in sequence and mediates the repression of the one expressed earlier.

#### *mlpt* RNAi expands *Kr* posteriorly and abolishes *gt* expression

**Fig. 4.**
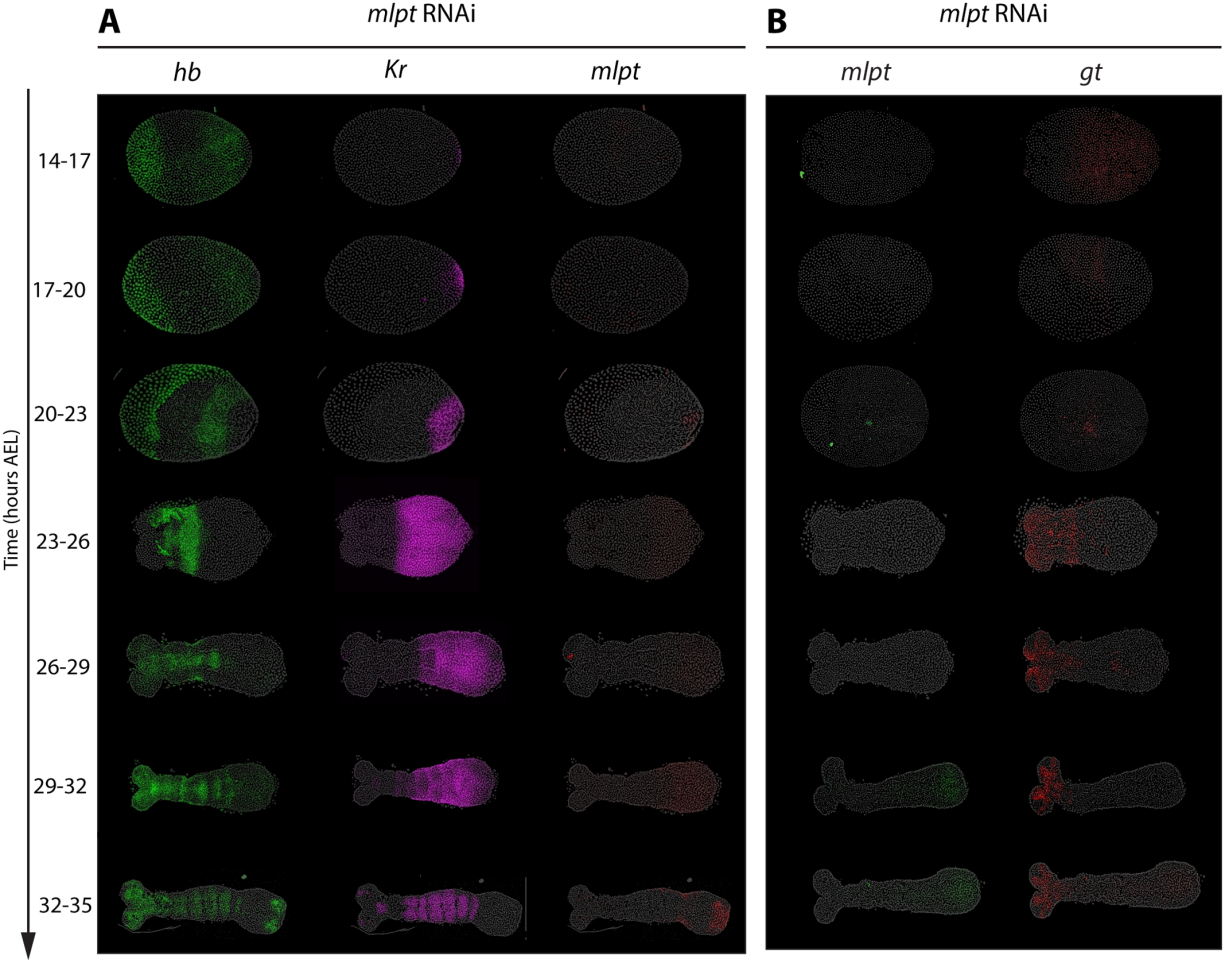
***mlpt* RNAi expands *Kr* posteriorly and abolishes *gt* expression.** Panels A and B show HCR FISH detecting subsets of the core gap genes (*hb*, *Kr*, *mlpt*, *gt*) following parental *mlpt* RNAi in staged embryos (14-35 h AEL). Panel A displays the expression patterns of *hb*, *Kr*, and *mlpt* in the same embryos. Panel B shows the expression of *mlpt* and *gt* in the same embryos. All embryos are oriented with anterior to the left and posterior to the right.

Embryos subjected to parental RNAi targeting *mlpt* showed (almost) complete loss of *mlpt* expression, indicating successful knockdown (Fig. 4A, red; Fig. 4B, green). Under this condition, *Kr* expression fails to clear from the posterior at 23-26 h AEL and 26-29 h AEL, where it does clear in the WT (compare with Fig. 1C,D), resulting in the extension of *Kr* expression significantly toward the posterior, where it remained broader compared to WT embryos, suggesting that *mlpt* normally represses *Kr* expression (Fig. 4A, magenta). In contrast, the trunk *gt* expression was completely absent in *mlpt* RNAi embryos (Fig. 4B), implying that *mlpt* is necessary for the activation of *gt*.

Notably, in *mlpt* RNAi embryos, *gt* failed to initiate altogether, rather than appearing posteriorly and then failing to stabilize, while *Kr* remained persistently expressed in the posterior instead of being cleared and later expanding more anteriorly during the stabilization phase. These findings confirm the regulatory role of *mlpt* within the gap gene network and support its position as an integral component of a genetic cascade that operates in the posterior during the initialization phase of gap gene expression, where it mediates both the activation of the next gene (*gt*) and the repression the preceding gene (*Kr*).

#### *gt* RNAi results in posterior expansion of *mlpt* expression

**Fig. 5.**
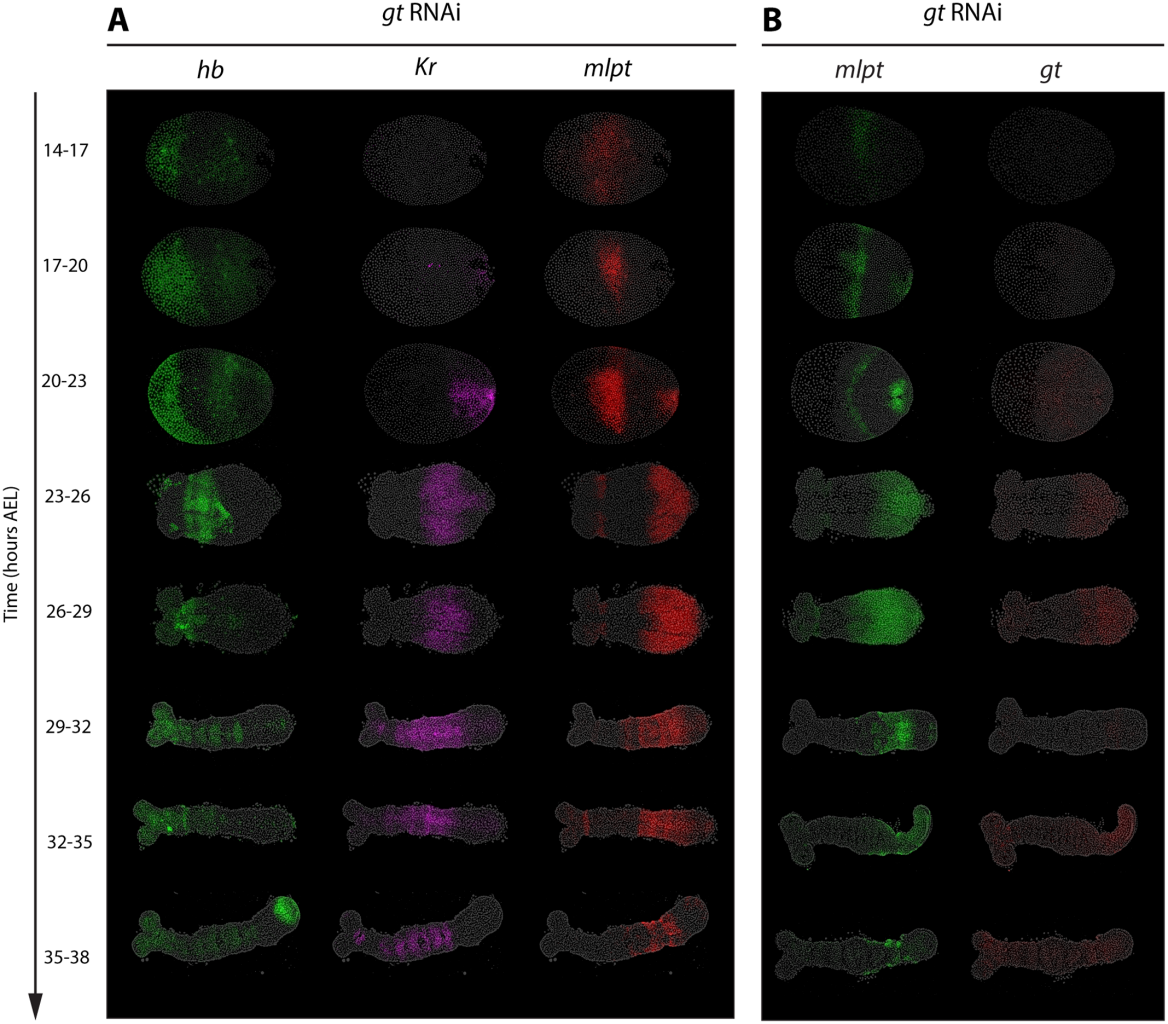
***gt* RNAi results in posterior expansion of *mlpt* expression.** Panels A and B show HCR FISH detecting subsets of the core gap genes (*hb*, *Kr*, *mlpt*, *gt*) following parental *gt* RNAi in staged embryos (14-38 h AEL). Panel A displays the expression of *hb*, *Kr*, and *mlpt*. Panel B shows the expression patterns of *mlpt* and *gt*. Embryos are oriented with posterior to the right in all panels.

In embryos subjected to *gt* RNAi, *gt* expression was reduced to background levels from 14 to 17 h AEL onward, confirming effective knockdown (Fig. 5B). Under these conditions, *mlpt* expression failed to clear from the posterior at either 23-26 h AEL or 26-29 h AEL, when it normally does in WT (Fig. 1C,D). As a result, *mlpt* persisted in the posterior and its domain expanded beyond normal boundaries (Fig. 5B, red; Fig. 5A, green), indicating that *gt* normally represses *mlpt*. Together, these observations identify *gt* as a negative regulator of *mlpt* within a genetic cascade that operates in the posterior during the initialization phase of gap gene expression.

### The role and regulation of *svb* within the gap genetic cascade

*svb* encodes a transcription factor best known in *Drosophila* for controlling epidermal differentiation, where it regulates the formation of cuticular trichomes (Payre et al., 1999; Chanut-Delalande et al., 2006). In its default state, Svb acts as a transcriptional repressor. Its activity is modulated by small peptides encoded by the *mlpt* locus, which bind Svb and induce a conformational change that converts it into a transcriptional activator. This Svb-Mlpt module is an evolutionarily conserved switch controlling diverse processes, from epidermal differentiation to embryonic segmentation. In *Tribolium*, both genes are expressed dynamically during early development, placing them in a position to function as part of the gap gene network (Ray et al., 2019).

#### Both *mlpt* and *svb* are required for *gt* expression

**Fig. 6.**
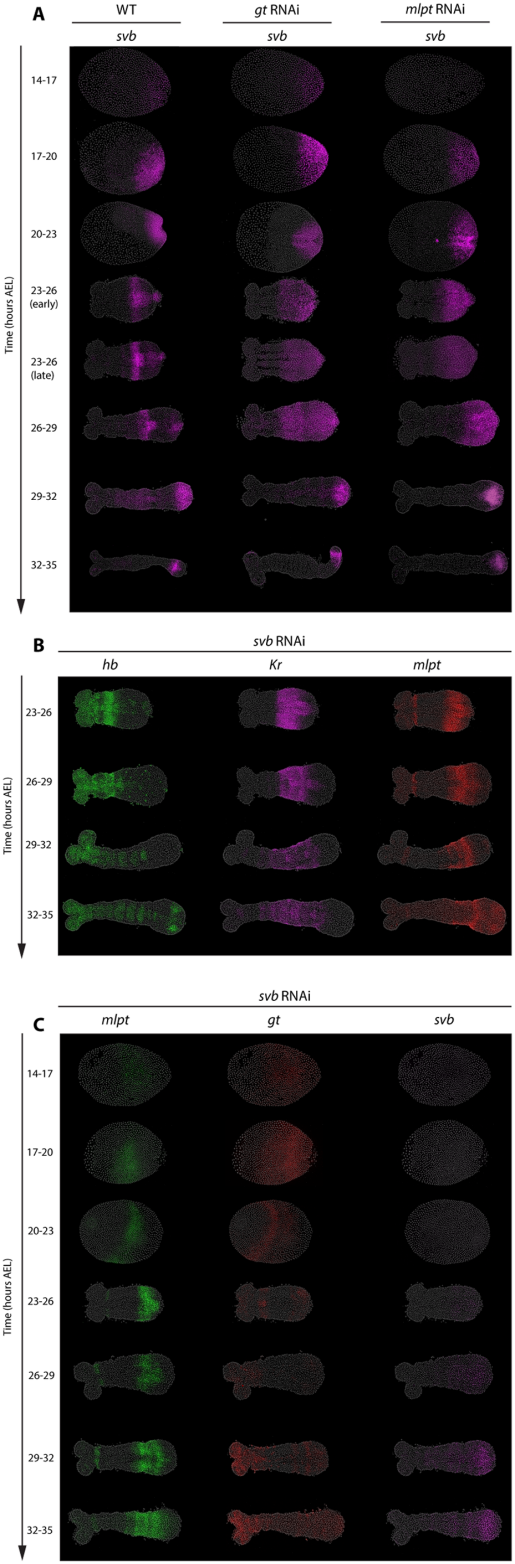
**The role and regulation of *svb* within the gap genetic cascade.** (A) Expression of *svb* in WT, *gt RNAi*, and *mlpt RNAi* embryos. (B) The expression patterns of *hb*, *Kr*, and *mlpt* following parental *svb* RNAi. (C) The expression of *mlpt*, *gt*, and *svb* following parental *svb* RNAi. Embryos were analyzed at various developmental stages (14-35 h AEL). All embryos are oriented with anterior to the left and posterior to the right.

In *Tribolium*, *svb* first appears as a posterior cap at 20-23 h AEL, then clears from the posterior, leaving behind a stripe of expression more anteriorly. It is subsequently re-expressed and maintained at the posterior through later patterning stages (Fig. 6A, *svb* in WT). Our data reveal a critical regulatory interaction between *svb* and *mlpt* during embryogenesis that is essential for proper gap gene expression. Consistent with the biochemical mechanism described in *Drosophila*, we find that both *mlpt* and *svb* are required to activate target genes, notably *gt*. RNAi experiments confirm this: embryos lacking either *mlpt* or *svb* fail to express *gt* (Fig. 4B, red; Fig. 6C, red). Consistent with this, the expression profiles of *Kr* and *mlpt* in *svb* RNAi embryos closely resemble those observed in *gt* RNAi (compare Fig. 6B to Fig. 5A,B): *Kr* expression is comparable to WT, whereas *mlpt* expression persists posteriorly.

These observations place *mlpt* and *svb* as cooperative components of the posterior genetic cascade, linking their inferred partnership to the regulation of early AP patterning genes.

#### *mlpt* mediates the transition from dynamic to static phases of *svb* expression

An unresolved question concerns the regulatory mechanisms controlling *svb* expression itself. As described above, *svb* expression initially emerges as a posterior cap at the blastoderm stage (20-23 h AEL), closely mirroring the expression domain of *cad* (Zhu et al., 2017; El-Sherif et al., 2014; Wolff et al., 1998; Schulz et al., 1998; Copf et al., 2004). Upon activation of *mlpt*, the posterior *svb* expression clears, and a distinct anterior expression band forms (Fig. 6A, WT). Crucially, in *mlpt* RNAi embryos, *svb* expression remains trapped within the posterior growth zone, failing to propagate anteriorly (Fig. 6A, *mlpt* RNAi). This highlights a critical role for *mlpt* in facilitating anterior propagation of *svb*. Importantly, *mlpt* cannot drive this transition on its own, since it does not encode a transcription factor; rather, it likely exerts its function through interaction with *svb*, enabling Svb to act as an activator, as established in *Drosophila*.

Thus, the most parsimonious model for stabilizing *svb* expression outside the growth zone involves the autoactivation of *svb* mediated by the Mlpt-bound Svb complex. For other genes, direct autoactivation is difficult to experimentally confirm, since knocking down the gene disrupts its expression entirely, obscuring any specific autoactivation link. However, in the case of *svb*, autoactivation is uniquely detectable through *mlpt* RNAi. Because *mlpt* is necessary for Svb's conversion into a coactivator, targeting *mlpt* selectively abolishes the autoactivation feedback loop without eliminating all *svb* expression, revealing the presence of this regulatory mechanism.

#### *gt* mediates the clearance of *svb* from the growth zone

In WT embryos, posterior clearance of *svb* occurs around 23-26 h AEL, a process likely mediated by repression from *gt* (Fig. 6A). In *gt* RNAi embryos, *svb* still propagates anteriorly out of the growth zone, presumably because *mlpt* expression remains intact, but it no longer clears from the posterior, leading to a persistent posterior *svb* domain (Fig. 6A). A similar lack of posterior clearance is observed in *Kr* and *hb* RNAi embryos (Fig. S1A,B), where *svb* propagates anteriorly but continues to be expressed in the posterior growth zone. Although *mlpt* is globally reduced in these knockdowns, a small anterior *mlpt* stripe persists, and this *mlpt* subdomain appears to be activated independently of the main gap gene cascade. This residual *mlpt* expression provides a plausible explanation for why *svb* propagation can still occur in *hb* and *Kr* RNAi embryos despite reduced *mlpt* levels overall.

Collectively, these results suggest that the anterior propagation of *svb* expression depends on the cooperative activity of Svb and Mlpt (presumably via the formation of a complex), which likely drives autoactivation of *svb* within a stabilizing GRN operating outside the growth zone. This is consistent with the GRN switching model, in which the stabilization phase is mediated by a static network that takes over from the dynamic cascade active in the posterior growth zone, thereby ensuring the transition from sequential activation to stable expression domains. This provides, however, a parsimonious explanation for the data, although confirming the underlying interactions requires further experimentation beyond the scope of this study.

### Computational modeling suggests GRN switching as a plausible basis for mediating gap gene expression patterns in *Tribolium*

**Fig. 7.**
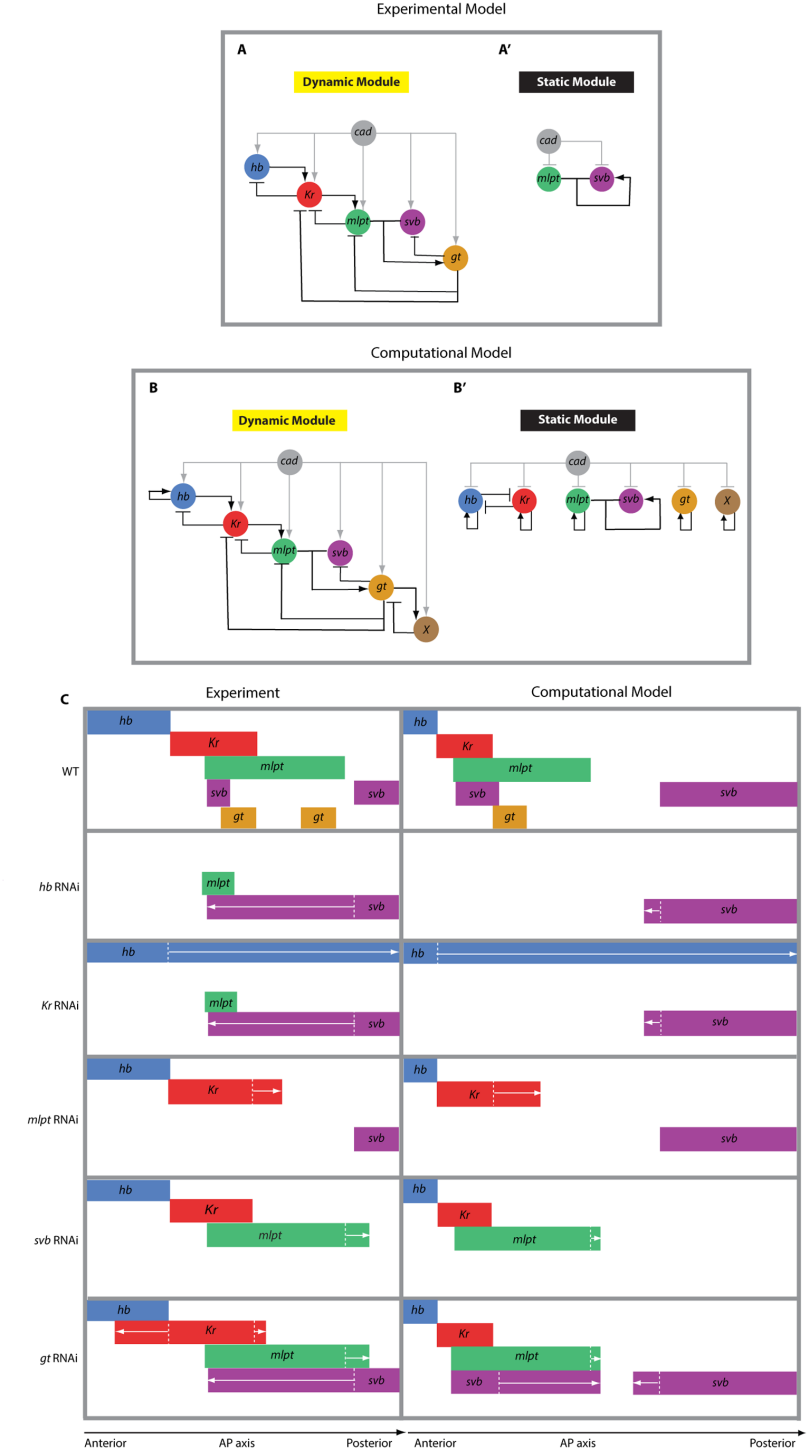
**Gap gene regulation and expression in experiments versus model.** (A,A′) Simple wiring schemes for the gap gene network, manually inferred from WT and RNAi phenotypes reported in this study. The network is shown separately for the posterior initiation phase (dynamic module; A) and the anterior stabilization phase (static module; A′). Only experimentally supported regulatory interactions are included. (B,B′) Computational models of the gap gene network based on the experimentally inferred schemes in (A,A′), supplemented with additional hypothetical interactions required to reproduce observed data. For the dynamic module (B), self-activation of *hb* and an additional unknown factor *X* interacting with *gt* were introduced. For the static module (B′), self-activation links were included for all genes except *svb* (the autoactivation of which via *mlpt* is experimentally supported), and factor *X* with a self-activation link was added. (C) Comparison between final expression patterns of gap genes in experiments (left) and in simulations (right). The experimental panel (left) is a schematic summary approximating the phenotypic findings observed in Figs 1-6, while the computational panel (right) depicts the final stable state (*t_final_*) of the *in silico* simulations. Note that the comparison between experimental findings and computational model results should focus on qualitative topological similarities (e.g. presence, absence, or extension of gene expression domains) rather than quantitative exactness.

To test whether our experimental observations are consistent with the GRN switching model, we developed a computational framework (Fig. 7B,B′) based on coupled ordinary differential equations (ODEs) that encode regulatory interactions inferred from our data (Fig. 7A,A′). The model includes the five gap genes analyzed in this study (*hb*, *Kr*, *mlpt*, *svb*, and *gt*), plus a putative regulator *X* introduced to account for posterior repression of *gt*. Importantly, it incorporates the Mlpt-Svb interaction, which our genetic experiments suggest is essential for activating *gt* and maintaining autoactivation of *svb*. While only *svb* autoactivation is directly supported experimentally, we extended this mechanism to other gap genes in the static GRN to test whether similar feedback could account for stable anterior domains. We note that the regulatory interactions depicted here, even those based on experimental evidence, may be indirect, as multiple network wirings can give rise to essentially the same dynamics. The aim of this modeling, instead, is to assess the plausibility of the overall mechanism (specifically, the GRN switching model) rather than to resolve the precise identity of every regulatory connection.

#### Computational model and *in silico* RNAi

The ODE-based model integrates activation, repression, and mRNA degradation dynamics (Dataset 1; Garcia-Guillen and El-Sherif, 2025). It contains a Cad-dependent dynamic GRN driving sequential activation in the posterior and a Cad-independent static GRN maintaining stabilized anterior domains. An *in silico* RNAi option sets expression of targeted genes to zero, enabling comparison to experimentally observed RNAi phenotypes. Fig. 7C presents a side-by-side comparison of the final stable state of the simulation (*t_final_*) against representative WT or RNAi embryos. This comparison is intended to highlight topological similarities (e.g. presence, absence, reduction, or extension of gene expression domains) rather than quantitative exactness. Consequently, minor discrepancies in domain width or exact intensity between the simulation and experiment are expected consequences of this qualitative approach. The experimental panel in Fig. 7C (left) provides a schematic summary of the phenotypic findings observed in Figs 1-6, while the computational model panel (right) depicts the final stable states derived from the *in silico* simulations (Movies 1-6).

#### WT simulation

In WT simulations (Fig. 7C; Movie 1), the model successfully reproduces the sequential activation of the gap genes *hb*, *Kr*, *mlpt*, and *gt*. Driven by the posterior-to-anterior gradient of Cad, these genes appear as dynamic waves that originate in the posterior and propagate anteriorly, mirroring the temporal sequence observed *in vivo*. As the cascade progresses, the model captures the transition of *svb* from a dynamic to a stable state. Initially, *svb* mirrors the posterior-to-anterior gradient of Cad. Subsequently, upon expression of *mlpt*, the formation of the Mlpt-Svb complex establishes a positive auto-regulatory loop that mediates the propagation of *svb* expression anteriorly, beyond the original posterior growth zone. Finally, the activation of *gt* at the end of the cascade leads to the repression of both *mlpt* and *svb* within the growth zone, resulting in their posterior clearance. Thus, the simulation captures the full logic of the system: a Cad-driven initialization cascade that generates sequential waves, followed by network-mediated stabilization and sharpening of spatial domains.

#### *in silico hb* and *Kr* RNAi phenotypes

The computational model successfully recapitulates the experimental phenotypes observed in *hb* and *Kr* RNAi embryos. When *hb* was knocked down *in silico* (Movie 2), expression of later-acting genes in the cascade (*Kr*, *mlpt*, and *gt*) failed to initiate in the posterior. This outcome matches our experimental findings (Fig. 7C) and confirms the role of *hb* as the initiating gene in the cascade.

Similarly, *in silico* knockdown of *Kr* reproduced the dual effect observed experimentally (Fig. 7C; Movie 3). The expression of *hb* expanded anteriorly, consistent with *Kr* normally repressing *hb*. At the same time, *mlpt* and *gt* expression failed to arise. Together, these simulations closely mirror the observed RNAi phenotypes and further validate the cascade wiring inferred from experimental perturbations.

#### *in silico* mlpt RNAi

Simulating RNAi targeting *mlpt* resulted in outcomes consistent with our RNAi experiments (Fig. 7C; Movie 4). Specifically, the simulation recapitulates the posterior expansion of *Kr* due to the loss of repression. Importantly, it also captures the eventual clearing of *Kr* at late stages; this occurs because *Kr* expression in our model relies on activation from the upstream gene *hb*, which itself ceases to be expressed due to repression by *Kr*. This demonstrates that the inherent logic of the genetic cascade is sufficient to explain late-stage *Kr* dynamics without invoking additional repressors.

With *mlpt* knocked down *in silico*, the absence of the *mlpt-svb* complex abolishes the positive feedback loop necessary for anterior propagation of *svb* expression. Consequently, in silico *svb* expression remains confined within the Cad-rich posterior growth-zone throughout development, precisely mirroring the experimentally observed phenotype in *mlpt* RNAi embryos (Fig. 7C). The modeling thus is in line with our interpretation that anterior propagation of *svb* relies specifically on the cooperative interaction between *mlpt* and *svb*, reinforcing the proposed regulatory role of *mlpt* within the GRN.

#### *In silico svb* RNAi

Simulating RNAi targeting *svb* (Fig. 7C; Movie 5) eliminates the Mlpt-Svb complex, thereby removing the activation of *gt*. As a result, *gt* fails to initiate. Because *gt* normally represses *mlpt*, its absence leads to a posterior expansion of the *mlpt* domain. Other gap genes (*hb* and *Kr*) retain their expected dynamics.

#### *In silico gt* RNAi

Simulated *gt* RNAi (Fig. 7C; Movie 6) further supports our experimental findings, revealing two key outcomes. First, the absence of *gt*-mediated repression results in prolonged persistence within the growth zone and the expansion of the *mlpt* expression domain towards posterior. Second, without posterior repression by *gt*, *svb* fails to clear from the growth zone but continues to propagate and stabilize anteriorly through the intact *mlpt-svb* auto-regulatory feedback loop. Thus, the computational *gt* RNAi scenario closely matches experimentally observed phenotypes, where the removal of *gt* leads to persistent expression of *mlpt* and *svb* in the posterior.

A noteworthy comparison arises between *svb* expression in *mlpt* versus *gt* RNAi conditions, observed both experimentally and in silico (Figs 6 and 7). In both cases, *svb* fails to clear from the posterior growth zone. However, the anterior dynamics differ: in *mlpt* RNAi embryos, *svb* is unable to propagate anteriorly, whereas in *gt* RNAi embryos, *svb* successfully propagates anteriorly but remains abnormally persistent in the posterior, producing an expanded expression domain. This distinction can be explained by the requirement of *mlpt* for anterior propagation of *svb*: in *gt* RNAi embryos, *mlpt* is still expressed, enabling propagation, while in *mlpt* RNAi embryos, its absence prevents anterior spread.

### Implications: computational results are consistent with a GRN switching framework

Our computational modeling suggests that the experimental phenotypes observed in *Tribolium* can be effectively explained by a GRN switching model. While the ‘general kinetic modulator’ model can, in principle, generate stable gene expression domains, it predicts that domain stability arises automatically from the decline of the kinetic modulator, independent of specific genetic interactions. This prediction appears inconsistent with our experimental findings regarding *svb*. In particular, the failure of *svb* to propagate anteriorly in *mlpt* RNAi embryos implies that stabilization likely requires the *mlpt*-mediated auto-activation, a feature the general kinetic modulator model does not explicitly account for. In contrast, the GRN switching framework, which incorporates a morphogen-dependent transition from a dynamic cascade to a static stabilizing network, successfully reproduces this behavior. Together, these results suggest that GRN switching offers a parsimonious explanation for the observed gap gene dynamics.

### Limitations of the study

While our data provide strong evidence for a genetic cascade driving the sequential activation of gap genes in the posterior, we acknowledge specific limitations regarding the mechanistic details of the proposed model. First, the involvement of the Mlpt-Svb complex in *Tribolium* is inferred from homology with *Drosophila* and functional outcomes, rather than demonstrated via direct biochemical interaction in this study. Consequently, our current genetic perturbation analysis captures the overarching regulatory logic (confirming that stabilization requires specific genetic inputs) but does not resolve the exhaustive molecular wiring. Therefore, we cannot currently distinguish between direct activation and indirect mechanisms (such as the repression of a repressor) that might underlie the observed genetic dependencies. Second, we focused our analysis on the core gap genes (*hb*, *Kr*, *mlpt*, *svb*, and *gt*) to delineate the fundamental sequential logic of the cascade. As a result, other established gap and gap-like genes such as *knirps* and *nubbin* were not included in our current model (Tidswell et al., 2021), although they likely contribute to the refinement and parallel processing of the network. Finally, although the failure of *svb* to propagate in *mlpt* RNAi embryos is consistent with the ‘GRN switching’ model, we have not yet isolated the specific genomic regulatory elements (enhancers) responsible for mediating this switch. Future work utilizing live imaging of enhancer activity will be required to definitively map the molecular logic that transitions gap genes from dynamic posterior initiation to stable anterior maintenance.

## DISCUSSION

Embryonic patterning is the process by which cells acquire distinct fates in precise spatial arrangements, enabling the formation of complex body plans. Classical frameworks, such as the French flag model, have emphasized spatial control, where morphogen gradients are interpreted by cells as positional information to establish domains of gene expression. More recently, however, evidence has accumulated that temporal dynamics also play a central role in pattern formation (Diaz-Cuadros et al., 2021; Palmeirim et al., 1997; Dale and Pourquié, 2000; Deschamps and Duboule, 2017; Verd et al., 2018; Pascoal et al., 2007; Pourquié, 2003; Sonnen et al., 2018; Soshnikova and Duboule, 2009; Briscoe and Small, 2015; Ebisuya and Briscoe, 2018; Sagner et al., 2021; Jaeger et al., 2004). In several developmental contexts, transcription factors are activated in a defined sequence over time, a logic termed temporal patterning (Filippopoulou and Konstantinides, 2024; Li et al., 2013). In neuroblast lineages, such temporal cascades diversify neuronal fates without directly mapping onto spatial order, because progenitors delaminate and migrate (Li et al., 2013; Kohwi and Doe, 2013; Averbukh et al., 2018; Simon and Konstantinides, 2021). By contrast, in other embryonic systems (including vertebrate Hox regulation, somitogenesis, and insect segmentation), temporal cascades appear to be translated directly into spatial registers, such that the order of gene activation in time is preserved in space (Diaz-Cuadros et al., 2021). This emerging concept, that embryonic patterning can involve a conversion of temporal order into spatial pattern, raises a central question: what molecular mechanisms allow dynamic gene regulatory programs to be ‘frozen’ into stable spatial domains?

We addressed this question using the gap gene network of *Tribolium castaneum* as a case study. Gap genes have been suggested to act in a genetic cascade, based on their sequential activation and on RNAi phenotypes that appeared consistent with cascade-like interactions. However, these earlier conclusions were drawn from static, late-stage expression patterns, when anterior domains were already stabilized. This left open the key question of whether a true cascade operates during the initial posterior activation phase. By directly tracking gap gene dynamics in WT and perturbed embryos, we show that gap genes do participate in a wiring-based genetic cascade during posterior initialization. This finding establishes the foundational regulatory logic shared by both prevailing mechanistic frameworks: the general kinetic modulation model, in which morphogens tune the speed of the cascade; and the GRN switching model, in which morphogens reconfigure regulatory interactions across the AP axis.

With the presence of an initiation cascade confirmed, the remaining challenge is to understand how dynamic activation transitions into stable spatial domains. Our results are consistent with a two-phase view of the system: an early dynamic cascade that drives posterior activation, followed by a later static GRN that maintains and stabilizes anterior expression domains as development proceeds.

The regulation of *svb* provides a particularly illustrative example of this transition (Fig. 6). In WT embryos, *svb* is activated in the posterior and subsequently propagates anteriorly upon *mlpt* expression. In *mlpt* knockdowns, this anterior propagation fails, indicating that stabilization in anterior regions requires specific regulatory interactions rather than arising solely from global kinetic effects. Computational modeling recapitulates these behaviors (Fig. 7) and shows that a morphogen-dependent shift in regulatory architecture can account for the observed phase-specific dynamics. This dynamic-to-static distinction also clarifies why general kinetic modulation may be insufficient by itself: while changes in transcription or decay rates could alter the overall pace of expression, they do not inherently explain why the same genes would operate first as a sequential cascade and later as a stabilizing network.

The GRN switching framework offers a parsimonious explanation for these observations, but we note that our data do not exclude the possibility that more complex forms of kinetic modulation (beyond those represented in our current model) could produce similar phenotypes. A definitive distinction between these mechanisms will require identifying the underlying regulatory elements and testing whether distinct enhancer logics indeed mediate the two phases of gap gene regulation.

This raises another important question: if the GRN switching scheme is correct, how can the same genes participate in two different networks: one dynamic at the posterior, the other static at the anterior? One plausible explanation is that each gene is controlled by distinct enhancers that encode its wiring within each GRN, a mechanism formalized in the ‘enhancer switching’ model (Zhu et al., 2017; Garcia-Guillen and El-Sherif, 2025). In this framework, every patterning gene possesses a ‘dynamic’ enhancer, which mediates its role in the posterior cascade, and a ‘static’ enhancer, which governs its stabilization in the anterior. Morphogen levels then determine the balance between these enhancers, promoting dynamic activity in the posterior and static activity in the anterior. The model successfully generates gene expression waves *in silico* (Zhu et al., 2017) and is in line with observations in *Drosophila* (El-Sherif and Levine, 2016; Hoermann et al., 2016) and vertebrate systems (Oginuma et al., 2010; Stauber et al., 2009). While promising, the model necessitates rigorous testing to determine if refinement or alteration is needed. An alternative possibility is enhancer pleiotropy (Preger-Ben Noon et al., 2018; Infante et al., 2015; Singh and Yi, 2021), in which a single enhancer encodes multiple regulatory logics, enabling a gene to participate in both phases through context-dependent outputs.

Recent technical advances make it possible to directly test these ideas in *Tribolium*. A framework combining assay for transposase-accessible chromatin with high-throughput sequencing with MS2-based live enhancer reporters has been developed to discover active enhancers and follow their dynamics *in vivo* (Mau et al., 2023). Initial results from this system have revealed candidate enhancers with dynamic-like and static-like activities, providing tentative support for the enhancer switching model, but stronger evidence will require systematic identification of enhancer pairs and functional tests through enhancer-specific deletions. Applying this framework to gap genes will be essential to determine whether their dual roles in posterior cascades and anterior stabilization are mediated by distinct enhancers or by pleiotropic regulatory logic.

Taken together, our findings provide direct evidence that gap genes in *Tribolium* function as a genetic cascade during the posterior initiation phase and show that aspects of their later behavior, particularly the regulation of *svb*, are consistent with a transition to a more stabilizing regulatory regime. These results suggest that GRN switching is a plausible framework for understanding how temporal activation sequences might contribute to spatial patterning, while also highlighting that additional mechanisms (such as more complex forms of kinetic modulation) cannot yet be excluded. A definitive distinction between these possibilities will require identifying the relevant regulatory elements and testing whether distinct enhancer activities underlie the two phases of gap gene regulation. More broadly, our work illustrates how combining high-resolution expression dynamics with perturbation analysis can clarify the relationship between temporal gene activation and spatial pattern formation, contributing to ongoing efforts to understand how timing and spatial information are integrated during embryogenesis.

## MATERIALS AND METHODS

### Beetle cultures

Beetle cultures (San Bernadino strain) were reared on flour supplemented with 5% dried yeast in a temperature- and humidity-controlled room at 24°C. To speed up development, beetles were reared at 32°C.

### Egg collections for developmental time windows

Developmental time windows of 3 h were generated by incubating 3 h egg collections at 24°C for the desired length of time. Beetles were reared in flour supplemented with 5% dried yeast. To assess phenotypic consistency and RNAi efficiency, an average of five embryos were inspected within each staged collection, and images of representative embryos were selected for presentation for each developmental stage.

### Fixation of embryos

*Tribolium castaneum* eggs were collected, floated off in 50% bleach (∼2 min), rinsed in water, and fixed at room temperature for 1 h in a two-phase formaldehyde/heptane mixture. Devitellinization was achieved by rapid shaking with methanol, followed by repeated passage through a 20-gauge needle. Embryos were washed three times in methanol and stored at −20°C.

### HCR fluorescence *in situ* hybridization (FISH)

Samples were rehydrated through 50% MeOH/PBT into PBT (1× PBS, 0.1% Tween-20), post-fixed in 4% formaldehyde/PBT for 30 min and rinsed in PBT. Embryos were pre-hybridized for 30 min in 30% probe hybridization buffer at 37°C, then incubated overnight with gene-specific HCR probes (Molecular Instruments) at 37°C. After four 15 min washes in 30% probe wash buffer (37°C) and three 5 min washes in 5× SSCT, snap-cooled fluorescent hairpins were applied in amplification buffer and allowed to react overnight at room temperature in the dark. Excess hairpins were removed by sequential washes in 5× SSCT, and embryos were mounted in SlowFade™ Gold (Thermo Fisher Scientific).

### Imaging and image processing

Confocal Z-stacks were acquired on Leica TCS SP5 and Nikon AX systems. Maximum-intensity projections were generated in Fiji/ImageJ.

### Use of generative AI

Generative AI tools (ChatGPT) were used to streamline the writing and presentation of the text and to assist with computer coding. The coding assistance involved an interactive process of code generation and refinement; however, all code was manually verified for accuracy by the authors.

### RNAi

Parental RNAi was performed to knockdown various gap genes. For dsRNA synthesis, a plasmid containing portions of a gap gene CDS flanked by T7 and T3 sites was used as template. T7 and T7-T3 primers were used to amplify the gap genes CDS fragment using the CloneAmp HiFi PCR Premix (Takara Bio). PCR cycle conditions were as follows: denaturation at 98°C for 15 s; annealing at 40°C (first five cycles)/55°C (remaining 25 cycles) for 15 s; elongation at 72°C for 1 min. The obtained PCR fragment was purified using the MinElute PCR Purification Kit (Qiagen). 1 µg of purified gap gene CDS served as template for dsRNA synthesis using the MEGAscript T7 Kit (Ambion). A gap gene dsRNA was diluted with injection buffer to a final concentration of 2 µg/µl and injected into female beetles. Coding sequence fragments used to generate the dsRNA probes in this study correspond to the fragments recommended by the iBeetle project (https://ibeetle-base.uni-goettingen.de/) for the respective genes.

### Computational modeling

The computational model used in this study is based on the GRN switching framework. A detailed description of the mathematical framework, governing equations, and underlying assumptions has been published previously (Zhu et al., 2017; Garcia-Guillen and El-Sherif, 2025). Briefly, the model simulates the concentration of gap gene products using coupled ODEs that account for mRNA production and decay. The specific regulatory logic (activation/repression interactions) and parameters used for the simulations in this study are provided in the MATLAB code included as Dataset 1. Parameters were selected to reproduce the qualitative spatiotemporal logic of the *Tribolium* gap gene network rather than optimized to fit experimental data.

## Supplementary Material

10.1242/biolopen.062391_sup1 Supplementary information
