# Supplementary material for: Spatiotemporal analysis of genetic perturbations reveals a genetic cascade driving Tribolium gap gene initialization
Source: Biol Open. 2026 Jan 19;15(1):bio062391. doi: 10.1242/bio.062391 (PMC12869515; doi:10.1242/bio.062391)
Supplement: Supplementary information [file biolopen-15-062391-s1.pdf]

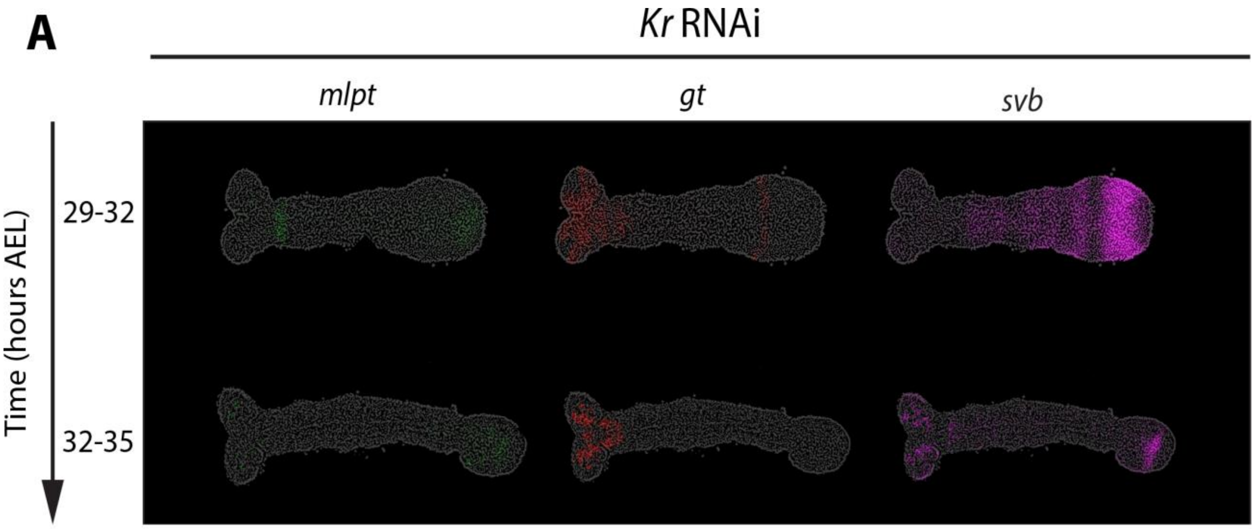

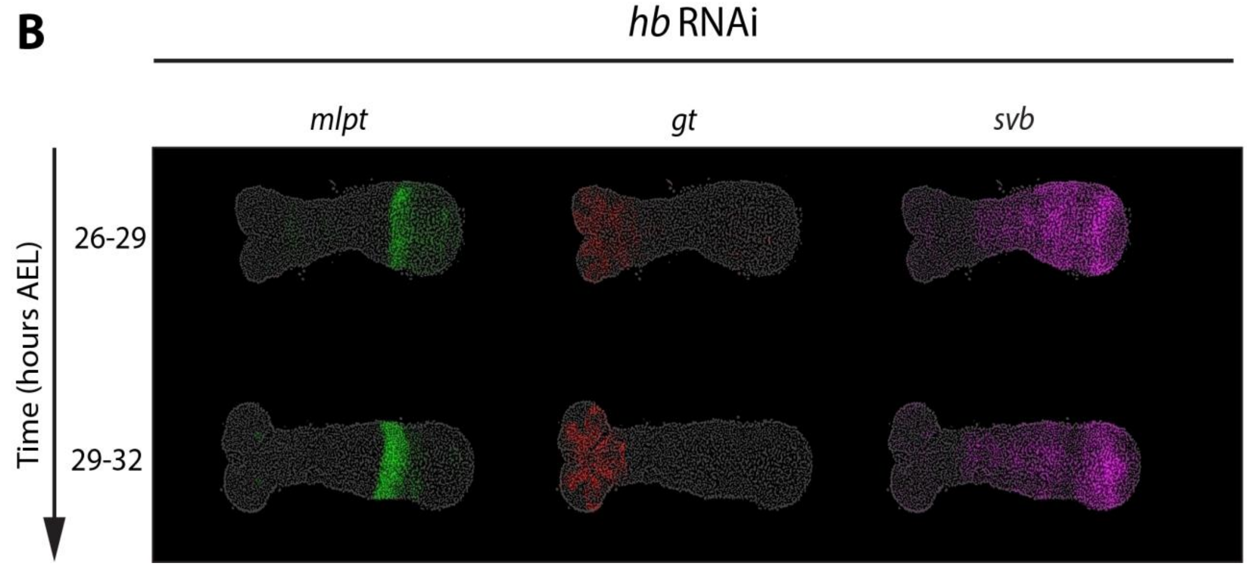

Fig. S1. *mlpt*, *gt*, and *svb* expressions in (A) *Kr* RNAi, and (B) *hb* RNAi embryos. All embryos are oriented with anterior to the left and posterior to the right.

Biology Open • Supplementary information

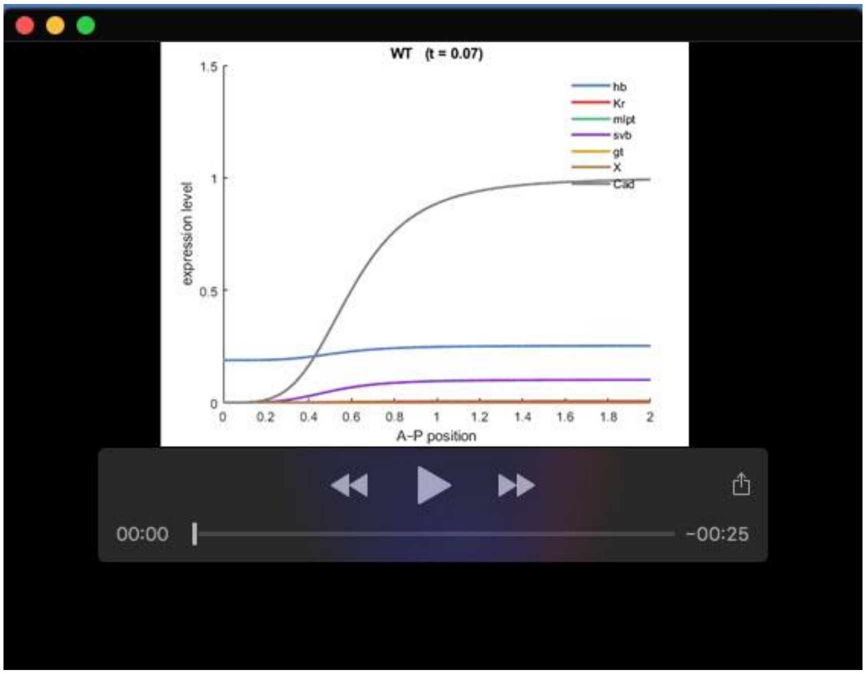

Movie 1. Wild-type simulation. Simulated spatiotemporal dynamics of gap gene expression in *Tribolium*. Sequential waves of *hb, Kr, mlpt,* and *gt* initiate in the posterior and propagate anteriorly. *svb* is activated independently in the posterior, propagates anteriorly with the aid of *mlpt*, and clears from the posterior through repression by *gt*, resulting in stable anterior domains.

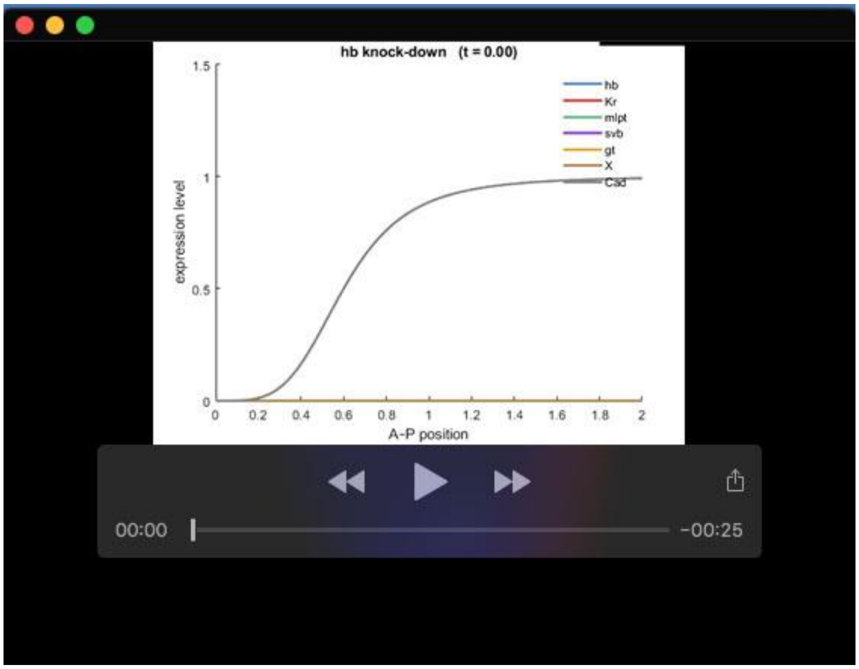

Movie 2. *hb* RNAi simulation. In silico knockdown of *hb*. *hb* expression is absent, and later cascade genes (*Kr, mlpt,* and *gt*) fail to initiate in the posterior. *svb* is still activated in the posterior, but without *mlpt* or *gt* activity it neither propagates anteriorly nor clears posteriorly, leaving it restricted to the posterior growth zone.

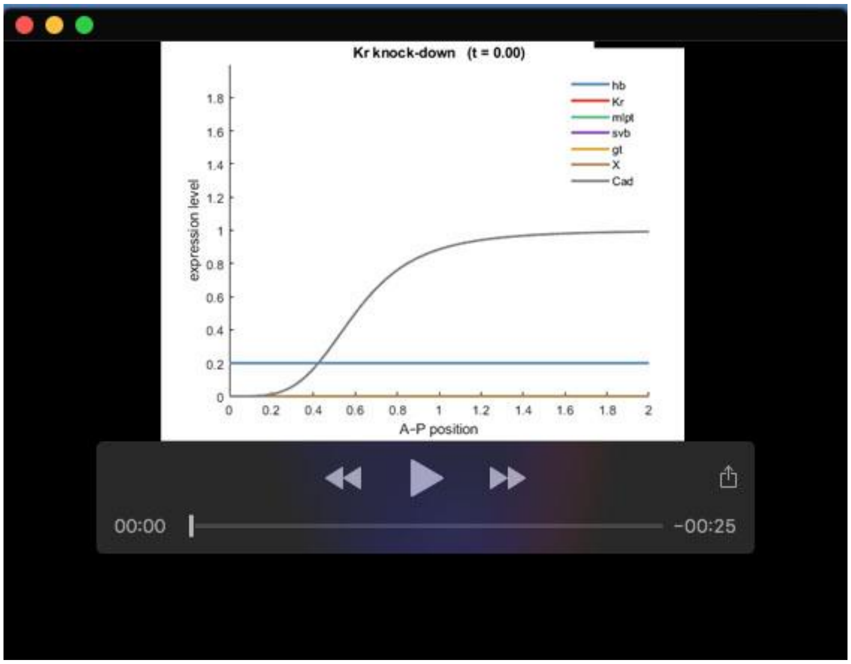

Movie 3. *Kr* RNAi simulation. In silico knockdown of *Kr*. *hb* expands anteriorly due to loss of repression. *mlpt* and *gt* fail to initiate. *svb* is activated in the posterior but cannot propagate anteriorly without *mlpt*, and also fails to clear posteriorly without *gt*, resulting in a persistent posterior cap.

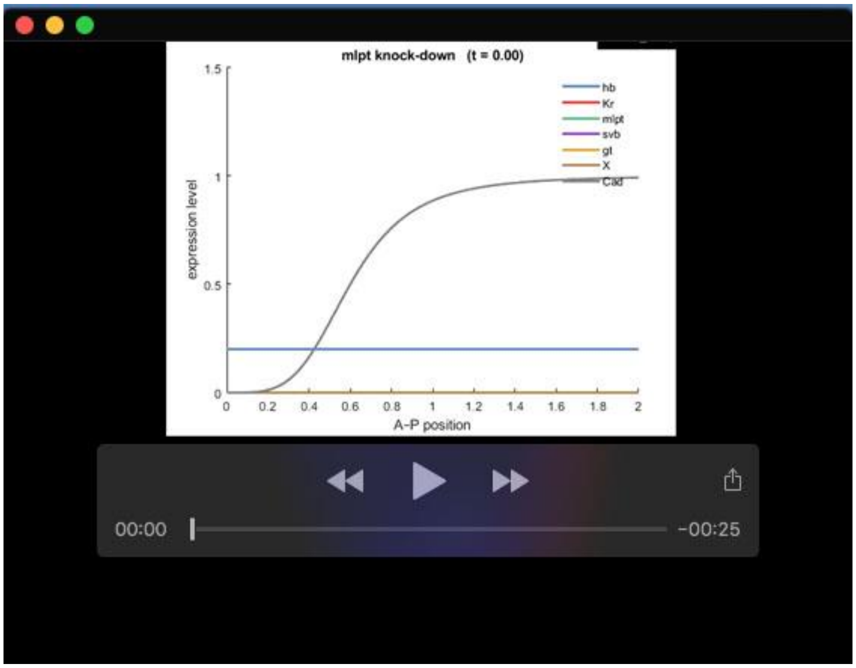

Movie 4. *mlpt* RNAi simulation. In silico knockdown of *mlpt*. *Kr* extends posteriorly, while *gt* fails to initiate. *svb* is activated in the posterior but fails to propagate anteriorly, demonstrating that its anterior spread depends on *mlpt*.

Biology Open • Supplementary information

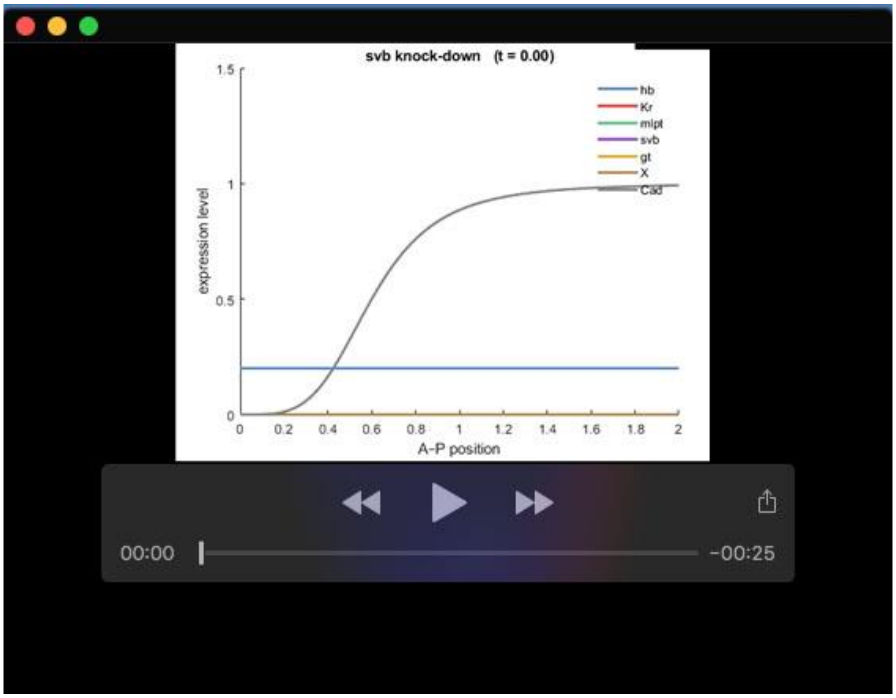

Movie 5. *svb* RNAi simulation. In silico knockdown of *svb. gt* fails to activate, reflecting the requirement of the Mlpt-Svb complex for *gt* expression, and *mlpt* expression expands due to the absence of repression by *gt*.

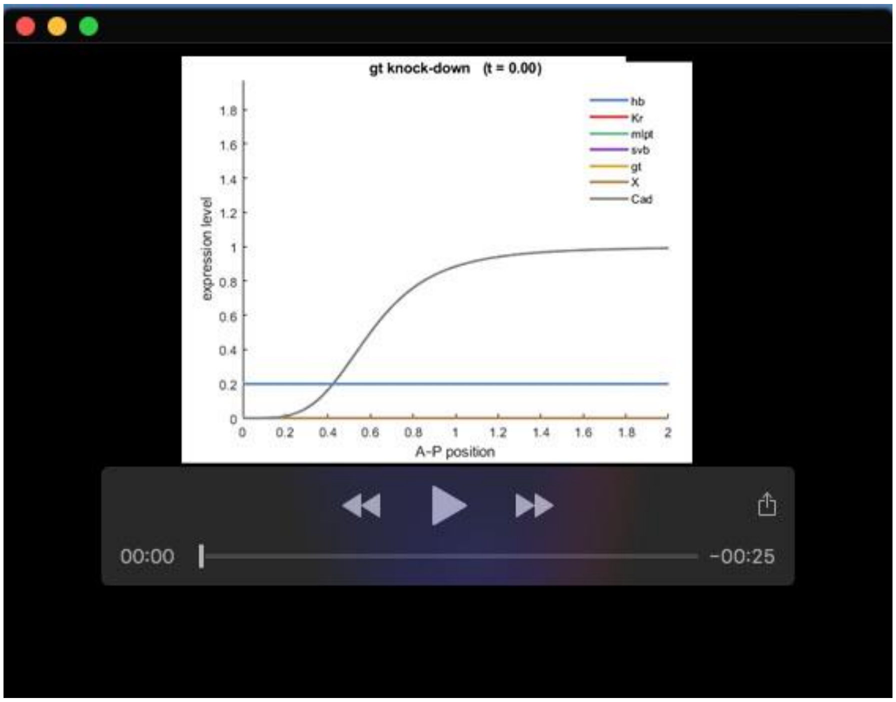

Movie 6. *gt* RNAi simulation. In silico knockdown of *gt. mlpt* expands posteriorly. *svb* is activated in the posterior and propagates anteriorly, but fails to properly clear from the posterior, resulting in an enlarged anterior domain.

Biology Open • Supplementary information

Dataset 1. Matlab implementation of the *Tribolium* gap gene GRN model. This file contains the Matlab script Tribolium_model_with_svb.m, which simulates the spatiotemporal dynamics of six gap genes (*hb, Kr, mlpt, svb, gt,* and a putative factor *X*) under the GRN switching framework. The model incorporates Cad-driven dynamic and static modules, as well as the Mlpt–Svb interaction required for anterior propagation and stabilization of *svb*. Users can simulate wild-type dynamics or knock down individual genes by modifying the parameter KD_gene. The script outputs two figures: (i) a time-resolved "movie" plot of expression waves across the AP axis (with optional export to .avi format), and (ii) a summary of stabilized expression domains at the end of development. All equations, parameters, and regulatory assumptions correspond to the computational framework described in the main text and Fig. 7.

```
function Tribolium_model_with_svb()
% ==========================================================================
% Tribolium_model_with_svb
% --------------------------------------------------------------------------
% WHAT THIS DOES
%   • Simulates a 1D Tribolium gap-gene GRN with 6 genes:
%       hb, Kr, mlpt, svb, gt, X
%     The model has Cad-driven dynamic/static modules and an mlpt·svb complex.
%   • Optionally "knocks down" one gene (clamps its level and derivative to 0).
%   • Produces two figures:
%       Figure 1: a movie-style line plot over time (6 genes + Cad gradient)
%                 with a proper legend and optional movie export.
%       Figure 2: a final-time "domain bars" summary (stacked vertical bands).
%
% HOW TO USE (no internals needed)
%   1) Choose a knock-down (or WT) by setting KD_gene below:
%        KD_gene = '';       % WT (no KD)
%        KD_gene = 'svb';    % knock down svb  (case-insensitive)
%        KD_gene = 'hb'|'Kr'|'mlpt'|'svb'|'gt'|'X';
%   2) Optional: set SAVE_MOVIE=true to export Figure 1 as an .avi.
%      The movie file is saved in your current folder (see 'pwd').
%   3) Run the file. Two figures will appear at the end:
%        • Figure 1 animates expression waves (with legend).
%        • Figure 2 shows stabilized domains as stacked colored bars.
%
% NOTES
%   • All equations, parameters, and color mapping follow your latest code.
%   • Colors: hb(steel-blue), Kr(red), mlpt(green), svb(purple),
%             gt(amber), X(brown #A87C4E), Cad(gray).
% ==========================================================================

% ————————————————————————— 0) HOUSEKEEPING ————————————————————————
clearvars; clc; close all;
```

Biology Open • Supplementary information

```
% —— 0.1) KNOCK-DOWN SELECTOR ('' for WT) ————————————————————————————————
% Valid names (case-insensitive): 'hb','Kr','mlpt','svb','gt','X'
KD_gene = '';            % examples: '' (WT), 'gt', 'svb', etc.

% Map KD name → index (1..6); 0 = no knock-down
gene_names = {'hb','Kr','mlpt','svb','gt','X'};
KD_idx     = find(strcmpi(KD_gene,gene_names),1);
if isempty(KD_idx);  KD_idx = 0;  end

% —— 0.2) MOVIE EXPORT (Figure 1) ————————————————————————————————————————
SAVE_MOVIE = true;                                 % set to true to save Figure 1
MOVIE_FILE = 'tribolium_gap_grn_svbRNAi.avi';
MOVIE_FPS  = 60;

% —— 0.3) FINAL-DOMAIN THRESHOLD (Figure 2) ——————————————————————————————
% A position belongs to a gene’s domain if >= DOMAIN_FRAC of that gene’s
% maximum at the final timepoint.
DOMAIN_FRAC = 0.30;

% ————————————————————————— 1) TIME & SPACE ——————————————————————————————
t_vec  = 0:0.01:7;           % developmental time
AP_vec = 0:0.001:2;          % antero-posterior axis

% ————————————————————————— 2) CAD GRADIENT (WT) —————————————————————————
peak       = 1;
flat_start = .6;
gn         = 4;
shift      = 0;
b2g_time   = 1;
v_wave     = .2;

% ————————————————————————— 3) INTEGRATE ALONG AP AXIS ———————————————————
n_genes   = 6;                                     % hb,Kr,mlpt,svb,gt,X
spacetime = zeros(n_genes, numel(t_vec), numel(AP_vec));

for p = 1:numel(AP_vec)
    % State vector: [hb Kr mlpt svb gt X   AP  b2g   flat    v]
    x0 = [0.2 0 0 0 0 0   AP_vec(p) b2g_time flat_start v_wave];
    if KD_idx~=0,  x0(KD_idx) = 0;  end       % KD starts at zero
    [~, sol] = ode45(@odefun, t_vec, x0);
    spacetime(:,:,p) = sol(:,1:n_genes).';
end

% ————————————————————————— 4) FIGURE 1: MOVIE LINES —————————————————————
% Fixed colors (6 genes + Cad), match your latest palette exactly
% hb, Kr, mlpt, svb, gt, X, Cad(legend only; actual Cad line uses last color)
color_order = [ ...
     86 128 193;  ... hb (steel-blue)
    229  51  50;  ... Kr (red)
     79 185 118;  ... mlpt (green)
    149  61 202;  ... svb (purple)
    219 152  40;  ... gt (amber)
    168 124  78;  ... X (brown #A87C4E)
    128 128 128]/256;  % Cad (gray)
```

Biology Open • Supplementary information

```
fig = figure('Color','w','Name','Figure 1: Gap GRN Movie');
axm = axes(fig); hold(axm,'on');

% Create persistent line handles with fixed colors & legend.
% Order in legend: hb, Kr, mlpt, svb, gt, X, Cad
legend_labels  = {'hb','Kr','mlpt','svb','gt','X','Cad'};
h = gobjects(7,1);
for i = 1:6
    h(i) = plot(axm, AP_vec, nan(size(AP_vec)), 'LineWidth', 2, ...
                'Color', color_order(i,:));
end
h(7) = plot(axm, AP_vec, nan(size(AP_vec)), 'LineWidth', 2, ...
            'Color', color_order(7,:)); % Cad

legm = legend(axm, h, legend_labels, 'Location','northeast'); % inside so visible
legm.Box = 'off';

ylim(axm, [0, max(1.5, max(spacetime(:)))]);
xlim(axm, [0, max(AP_vec)]);
xlabel(axm, 'A-P position'); ylabel(axm, 'expression level');

% Optional movie setup
if SAVE_MOVIE
    vw = VideoWriter(MOVIE_FILE); vw.FrameRate = MOVIE_FPS; open(vw);
end

for k = 1:numel(t_vec)
    % Update the six gene traces (rows 1..6 of spacetime)
    gp = squeeze(spacetime(:,k,:));       % [6 x numel(AP_vec)]
    for i = 1:6
        set(h(i), 'YData', gp(i,:));
    end
    % Update Cad
    cad_prof = cad_gradient(AP_vec, t_vec(k), b2g_time, flat_start, v_wave);
    set(h(7), 'YData', cad_prof);

    % Title per frame
    if KD_idx==0
        ttl = sprintf('WT   (t = %.2f)', t_vec(k));
    else
        ttl = sprintf('%s knock-down   (t = %.2f)', gene_names{KD_idx}, t_vec(k));
    end
    title(axm, ttl);

    drawnow;

    if SAVE_MOVIE
        writeVideo(vw, getframe(fig));
    end
end

if SAVE_MOVIE
    close(vw);
    fprintf('Movie saved to: %s\n', fullfile(pwd, MOVIE_FILE));
```

Biology Open • Supplementary information

```
end

% ─────────────────────────── 5) FIGURE 2: DOMAIN BARS ────────────────────
% Shows stabilized domains at final time as stacked vertical colored bars.
k_end = numel(t_vec);
final_profiles = squeeze(spacetime(:,k_end,:));   % [6 x numel(AP_vec)]

barfig = figure('Color','w','Name','Figure 2: End-of-simulation domains');
ax = axes(barfig); hold(ax,'on');

% Top→bottom rows: hb, Kr, mlpt, svb, gt, X
row_height = 1;                     % thickness of each row
y_top      = n_genes * row_height;   % y at the top row (hb)

for gi = 1:n_genes
    prof = final_profiles(gi,:);
    maxval = max(prof);
    if maxval == 0
        mask = false(size(prof));
    else
        mask = (prof >= DOMAIN_FRAC * maxval);
    end

    % Draw contiguous “true” segments as filled rectangles
    dmask  = diff([false, mask, false]);
    starts = find(dmask == 1);
    ends_  = find(dmask == -1) - 1;

    % Vertical span for this gene's row — rows TOUCH vertically
    y1 = y_top - (gi-1)*row_height;   % top edge of this row
    y0 = y1   - row_height;           % bottom edge (no gap)

    for seg = 1:numel(starts)
        x1 = AP_vec(starts(seg));
        x2 = AP_vec(ends_(seg));
        patch('XData',[x1 x2 x2 x1], 'YData',[y0 y0 y1 y1], ...
              'FaceColor', color_order(gi,:), 'EdgeColor','none', ...
              'FaceAlpha', 1.0, 'Parent', ax);
    end
end

% Axes cosmetics (YTick must be increasing)
ax.YLim = [0, n_genes*row_height];
ax.XLim = [min(AP_vec) max(AP_vec)];

% Tick centers for touching rows, bottom→top order
y_centers_desc = (y_top - (0:n_genes-1)*row_height) - 0.5*row_height;
y_centers = fliplr(y_centers_desc);
ax.YTick = y_centers;
ax.YTickLabel = fliplr(gene_names);   % bottom label 'X', top label 'hb'
ax.YDir = 'normal';

xlabel(ax,'A–P position');
ylabel(ax,'genes (top→bottom)');
title(ax, sprintf('Stabilized expression domains at t = %.2f', t_vec(k_end)));
```

Biology Open • Supplementary information

```
% Legend built from colored dummy patches (robust across MATLAB versions)
leg_handles = gobjects(n_genes,1);
for gi = 1:n_genes
    leg_handles(gi) = patch(ax, [NaN NaN NaN], [NaN NaN NaN], color_order(gi,:), ...
                        'EdgeColor','none', 'HandleVisibility','on', ...
                        'Visible','off');
end
lgd = legend(ax, leg_handles, gene_names, 'Location','northeastoutside');
lgd.Box = 'off';

% ========================================================================
%                           NESTED FUNCTIONS
% ========================================================================
    function dx = odefun(t,x)
        % State unpacking
        hb   = x(1);  Kr   = x(2);  mlpt = x(3);
        svb  = x(4);  gt   = x(5);  X    = x(6);
        AP   = x(7);  b2g  = x(8);  flat = x(9);  v = x(10);

        % Enforce knock-down (value + derivative → 0)
        if KD_idx~=0
            switch KD_idx
                case 1, hb   = 0;
                case 2, Kr   = 0;
                case 3, mlpt = 0;
                case 4, svb  = 0;
                case 5, gt   = 0;
                case 6, X    = 0;
            end
        end

        % Cad morphogen (blastoderm: static; germband: retracting)
        g = cad_gradient(AP, t, b2g, flat, v);

        % Constants (unchanged from your model)
        s = .4;  n = 5;  lambda = 1;  c = 1;  d = 3;

        % mlpt·svb complex (mass-action product)
        mlpt_svb = mlpt * svb;

        % Dynamic enhancers (Cad ON)
        dyn(1) = g/(1+g) * (hb/(s*.5))^n /(1+(hb/(s*.5))^n) ...
                         * 1/(1+(Kr/(s*.3))^n);                          % hb
        dyn(2) = g/(1+g) * (hb/s)^n      /(1+(hb/s)^n)      ...
                         * 1/(1+(mlpt/0.25)^n)              ...
                         * 1/(1+(gt/0.01)^n);                              % Kr
        dyn(3) = g/(1+g) * (Kr/(.8*.5))^n/(1+(Kr/(.8*.5))^n) ...
                         * 1/(1+(gt/0.3)^n);                              % mlpt
        dyn(4) = g/(1+g) * 1/(1+(gt/0.01)^n);                             % svb
        dyn(5) = 2*g/(1+g) * (mlpt_svb/0.5)^n /(1+(mlpt_svb/0.5)^n) ...
                         * 1/(1+(X/0.1)^n);                               % gt
        dyn(6) = g/(1+g) * (gt/s)^n /(1+(gt/s)^n);                        % X

        % Static enhancers (Cad OFF)
```

Biology Open • Supplementary information

```
        stat(1) = 1/(1+g) * (hb/s)^n   /(1+(hb/s)^n)   / (1+(Kr/s)^n);
        stat(2) = 1/(1+g) * (Kr/s)^n   /(1+(Kr/s)^n)   / (1+(hb/s)^n);
        stat(3) = 1/(1+g) * (mlpt/s)^n /(1+(mlpt/s)^n);
        stat(4) = 1/(1+g) * (mlpt_svb/s)^n /(1+(mlpt_svb/s)^n);             % svb
        stat(5) = 1/(1+g) * (gt/s)^n   /(1+(gt/s)^n);
        stat(6) = 1/(1+g) * (X/s)^n    /(1+(X/s)^n);

        % ODEs (apply KD: derivative = 0 for the chosen gene)
        genes = [hb Kr mlpt svb gt X];
        dx    = zeros(10,1);
        for gIdx = 1:6
            if KD_idx==gIdx
                dx(gIdx) = 0;
            else
                dx(gIdx) = c*stat(gIdx) + d*dyn(gIdx) - lambda*genes(gIdx);
            end
        end
        dx(7:10) = 0;   % AP, b2g, flat, v are parameters (no dynamics)
    end

    function g = cad_gradient(AP_pos, t_now, b2g, flat, v)
        % Cad: static in blastoderm; then steeper and retracting in germband
        if t_now < b2g
            n_eff = gn;  flat_len = flat;
        else
            n_eff = min(gn*exp(t_now - b2g),100);
            flat_len = flat + v*(t_now - b2g);
        end
        z = (AP_pos - shift)/flat_len;
        g = peak * (z.^n_eff ./ (1+z.^n_eff));
        g(AP_pos - shift < 0) = 0;
    end
end
```
